# Clinical risk predictors in atrial fibrillation patients following successful coronary stenting: ENTRUST-AF PCI sub-analysis

**DOI:** 10.1007/s00392-020-01760-4

**Published:** 2020-10-24

**Authors:** Andreas Goette, Lars Eckardt, Marco Valgimigli, Thorsten Lewalter, Petra Laeis, Paul-Egbert Reimitz, Rüdiger Smolnik, Wolfgang Zierhut, Jan G. Tijssen, Pascal Vranckx

**Affiliations:** 1 Medizinische Klinik II: Kardiologie Und Intensivmedizin, St. Vincenz-Krankenhaus, Am Busdorf 2, 33098 Paderborn, Germany; 2 Working Group of Molecular Electrophysiology, University Hospital Magdeburg, Magdeburg, Germany; 3 Atrial Fibrillation Network, Munster, Germany; 4 Division of Electrophysiology, Department of Cardiology and Angiology, University of Munster, Munster, Germany; 5 Department of Cardiology, Inselspital, Bern University Hospital, University of Bern, Bern, Switzerland; 6 Department of Cardiology, Hospital Munich South, Munich, Germany; 7 University of Bonn, Bonn, Germany; 8 Daiichi Sankyo Europe, Munich, Germany; 9 Department of Cardiology, Amsterdam University Medical Centers, University of Amsterdam, Amsterdam, The Netherlands; 10 Cardialysis, Rotterdam, The Netherlands; 11 Department of Cardiology and Intensive Care, Jessa Ziekenhuis, Faculty of Medicine and Life Sciences at the Hasselt University, Hasselt, Belgium

**Keywords:** Atrial fibrillation, Coronary stenting, NOACs, CHA_2_DS_2_-VASc, Edoxaban

## Abstract

**Aims:**

This subgroup analysis of the ENTRUST-AF PCI trial (ClinicalTrials.gov Identifier: NCT02866175; Date of registration: August 2016) evaluated type of AF, and CHA_2_DS_2_-VASc score parameters as predictors for clinical outcome.

**Methods:**

Patients were randomly assigned after percutaneous coronary intervention (PCI) to either edoxaban (60 mg/30 mg once daily [OD]; *n* = 751) plus a P2Y_12_ inhibitor for 12 months or a vitamin K antagonist [VKA] (*n* = 755) plus a P2Y_12_ inhibitor and aspirin (100 mg OD, for 1–12 months). The primary outcome was a composite of major/clinically relevant non-major bleeding (CRNM) within 12 months. The composite efficacy endpoint consisted of cardiovascular death, stroke, systemic embolic events, myocardial infarction (MI), and definite stent thrombosis.

**Results:**

Major/CRNM bleeding event rates were 20.7%/year and 25.6%/year with edoxaban and warfarin, respectively (HR [95% CI]: 0.83 [0.654–1.047]). The event rates of composite outcome were 7.26%/year and 6.86%/year, respectively (HR [95% CI]): 1.06 [0.711–1.587]), and of overall net clinical benefit were 12.48%/year and 12.80%/year, respectively (HR [(95% CI]: 0.99 [(0.730; 1.343]). Increasing CHA_2_DS_2_-VASc score was associated with increased rates of all outcomes. CHA_2_DS_2_-VASc score ≥ 5 was a marker for stent thrombosis. Paroxysmal AF was associated with a higher occurrence of MI (4.87% versus 2.01%, *p* = 0.0024).

**Conclusion:**

After PCI in AF patients, increasing CHA_2_DS_2_-VASc score was associated with increased bleeding rates and CHA_2_DS_2_-VASc score (≥ 5) predicted the occurrence of stent thrombosis. Paroxysmal AF was associated with MI. These findings may have important clinical implications in AF patients.

## Introduction

Around 15% of patients with atrial fibrillation (AF) have associated coronary artery disease which may require percutaneous coronary intervention (PCI) [1]. In these patients, oral anticoagulation is necessary for stroke prevention and antiplatelet therapy to prevent stent thrombosis, myocardial infarction (MI), and the need for urgent repeat revascularization all of which need to be balanced against the increased risk of bleeding [2]. Overall, it remains unclear how clinical outcome after PCI can be predicted in AF patients.

The optimal combination of novel anticoagulant agents, as well as the duration of treatment, for patients with acute coronary syndrome (ACS) or elective PCI along with coexisting AF have been studied in four randomised clinical trials [2–5]. Although the 2017 ESC focussed update on dual antiplatelet therapy recommended a triple-therapy strategy as short as possible (consisting of an oral anticoagulant, aspirin 75–100 mg, and clopidogrel 75 mg once daily) in AF patients presenting with ACS and/or undergoing PCI [6], studies have shown an increase in the absolute risk of major haemorrhage associated with co-prescription of an oral anticoagulant with antiplatelet therapy, in particular triple therapy [2–5, 7, 8].

Findings from non-vitamin K antagonist oral anticoagulant (NOAC) trials [2–5] have reported reduced bleeding events in patients receiving dual antithrombotic therapy (DAT) combining an NOAC and a P2Y_12_ inhibitor versus triple antithrombotic therapy (TAT) consisting of a vitamin K antagonist (VKA), a P2Y_12_ inhibitor, and aspirin.

The ENTRUST-AF PCI study (NCT02866175) was a randomised, multicentre, open-label, non-inferiority phase 3b trial with masked outcome evaluation at 186 sites in 18 countries [2]. In this trial, the edoxaban-based regimen was non-inferior for bleeding compared with the VKA-based regimen, without significant differences in ischaemic events [2].

Although the CHA_2_DS_2_-VASc score is used and validated in AF patients [9, 10], there is limited evidence for validity of the CHA_2_DS_2_-VASc score in patients with AF following PCI [11]. Therefore, it remains unclear if the score is useful to predict bleeding and ischaemic outcomes in this subset of patients. Of note, the highest prevalence of left atrial thrombi has been reported in AF patients aged ≥ 75 years combined with a history of congestive heart failure (CHF), whereas ventricular ischaemia appears to be related to paroxysmal AF [10, 12]. Thus, these specific sub-cohorts of AF patients may also have high adverse event rates after PCI.

The present subgroup analysis of the ENTRUST-AF PCI trial evaluated CHA_2_DS_2_-VASc score, age, history of CHF, and type of AF as potential determinants for the risk of bleeding, MACE (including MI) and any stent thrombosis, as well as for net clinical benefit.

## Methods

In the ENTRUST-AF PCI trial, patients with AF were investigated after successful coronary stenting. The study design and primary trial results were previously described in more detail [2, 13]. In brief, patients were randomly assigned (1:1) from 4 h to 5 days after PCI to either edoxaban (60 mg once daily, or dose reduced to 30 mg per day; *n* = 751) plus a P2Y_12_ inhibitor for 12 months or a VKA (*n* = 755) in combination with a P2Y_12_ inhibitor and aspirin (100 mg once daily, for 1–12 months). The dose of edoxaban could be reduced by 50% in patients with the following clinical characteristics: creatinine clearance 15–50 mL/min, bodyweight ≤ 60 kg, or concomitant use of specified potent P-glycoprotein inhibitors (cyclosporine, dronedarone, erythromycin, or ketoconazole).

The primary outcome was a composite of major or clinically relevant non-major (CRNM) bleeding within 12 months. The primary efficacy outcome was the composite of cardiovascular death, stroke, systemic embolic events (SEE), MI, and definite stent thrombosis. Other secondary outcomes include net clinical benefit, defined as the composite of cardiovascular disease, stroke, SEE, spontaneous MI, definite stent thrombosis, and International Society on Thrombosis and Haemostasis (ISTH) defined major bleeding. Any stent thrombosis is defined as a composite of definite, probable, and possible stent thrombosis (as per ARC consensus definitions). Definite stent thrombosis was a secondary endpoint, while statistical analysis of any stent thrombosis was performed post hoc. All suspected endpoint events were adjudicated by an independent Clinical Events Committee in a blinded manner and without any knowledge of the subject’s assigned treatment regimen [2, 13].

Patients were categorised by age groups (< 75 years / ≥ 75 years), presence or absence of history of CHF, and type of AF (paroxysmal, persistent, long-standing persistent and permanent; definition according to 2016 ESC/EACTS Guidelines for the management of atrial fibrillation [7]) to assess the effects on primary and secondary outcomes in the overall population and separately in patients receiving edoxaban and warfarin. All statistical analyses should be interpreted in a purely descriptive exploratory way.

Similar to post hoc landmark analyses on primary parameters, a post hoc landmark analysis was performed with a landmark at Day 14 regarding the net clinical benefit. The landmark at 14 days was selected based on the international normalised ratio distribution over time and inspection of the Kaplan–Meier curve.

The study was conducted in accordance with the Declaration of Helsinki, International Conference on Harmonisation guidelines on Good Clinical Practice (ICH E6), and applicable regulatory requirements. The final study protocol and informed consent form were reviewed and approved by the ethics boards/institutional review boards and corresponding health authorities for all participating study sites.

## Results

Overall, 1,506 patients were randomised after a median time from PCI to randomisation of 45.1 h (interquartile range [IQR] 22.2–76.2). Major or CRNM bleeding event rates were 20.7%/year and 25.6%/year in patients receiving edoxaban and warfarin, respectively (hazard ratio [HR] [95% confidence interval (CI)]): 0.83 [(0.654–1.047]). The event rates of primary composite outcome of cardiovascular death, stroke, SEE, MI, and definite stent thrombosis were 7.26%/year and 6.86%year in patients receiving edoxaban and warfarin, respectively (HR [95% CI]): 1.06 [0.711–1.587]).

### CHA_2_DS_2_-VASc score

**Fig. 1:**
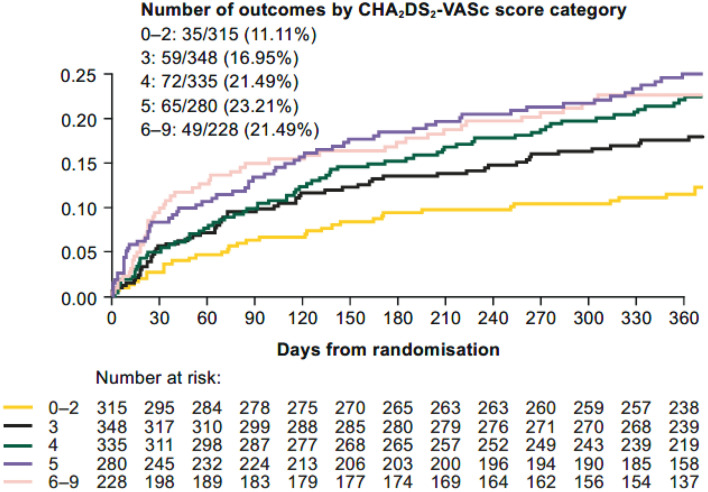
Time to first major or CRNM bleeding by CHA_2_DS_2_-VASc category. *CRNM* clinically relevant non-major

**Fig. 2:**
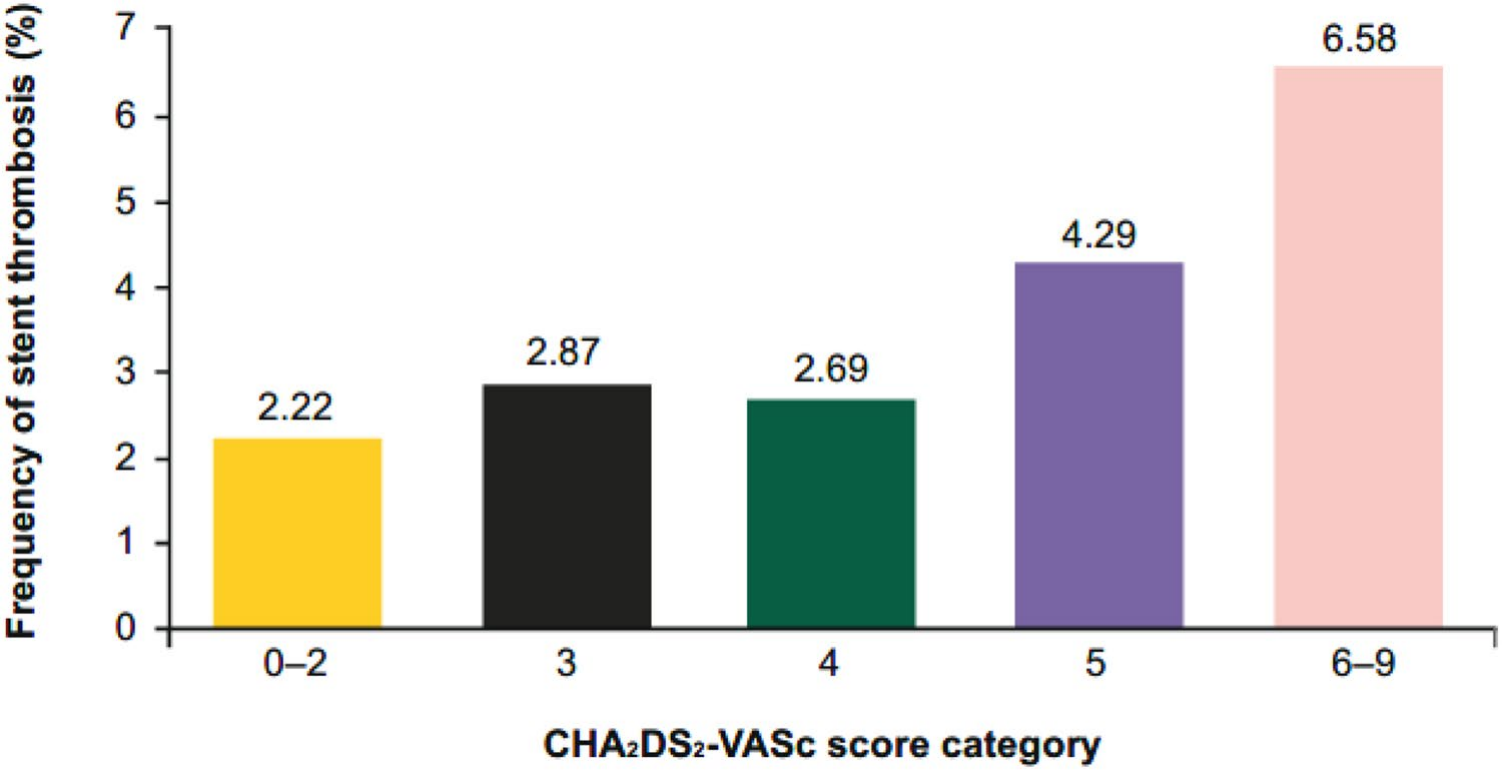
Frequency of any stent thrombosis by CHA_2_DS_2_-VASc score category

In patients categorised by CHAD_2_DS_2_-VASC score (0–2, 3, 4, 5, 6–9), increasing CHA_2_DS_2_-VASc score was associated with increased major or CRNM bleeding (Fig. 1). Similar to the CHA_2_DS_2_-VASc score, HAS-BLED score predicted bleeding (Table 1). Increasing CHA_2_DS_2_-VASc score was also associated with increased events of primary efficacy and net clinical benefit (Table 2). The effect of edoxaban versus VKA was consistent independent of the CHA_2_DS_2_-VASc score. The event rates of definite (14 cases) and probable (9 cases) stent thromboses were very low [2]. Of note, CHA_2_DS_2_-VASc score ≥ 5 was a marker for occurrence of any stent thrombosis during follow-up (Fig. 2).

**Table 1:** Frequency of primary composite outcome (major or CRNM bleeding [ISTH]) by HAS-BLED score category

HAS-BLED score category	Primary composite outcome	
	Events/*N*	%
missing	12/93	12.90
1–2	62/443	14.00
3	141/708	19.92
4–7	65/262	24.81

The HAS-BLED score has only been calculated if all nine items were non-missing

**Table 2:** Frequency of primary composite outcome and net clinical benefit by CHA_2_DS_2_-VASc score category

CHA_2_DS_2_-VASc score category	Primary composite outcome		Net clinical benefit	
	Events/*N*	%	Events/*N*	%
0–2	12/315	3.81	17/315	5.40
3	19/348	5.46	30/348	8.62
4	23/335	6.87	50/335	14.93
5	20/280	7.14	35/280	12.50
6–9	21/228	9.21	33/228	14.47

### Age, history of CHF, and AF type

**Fig. 3:**
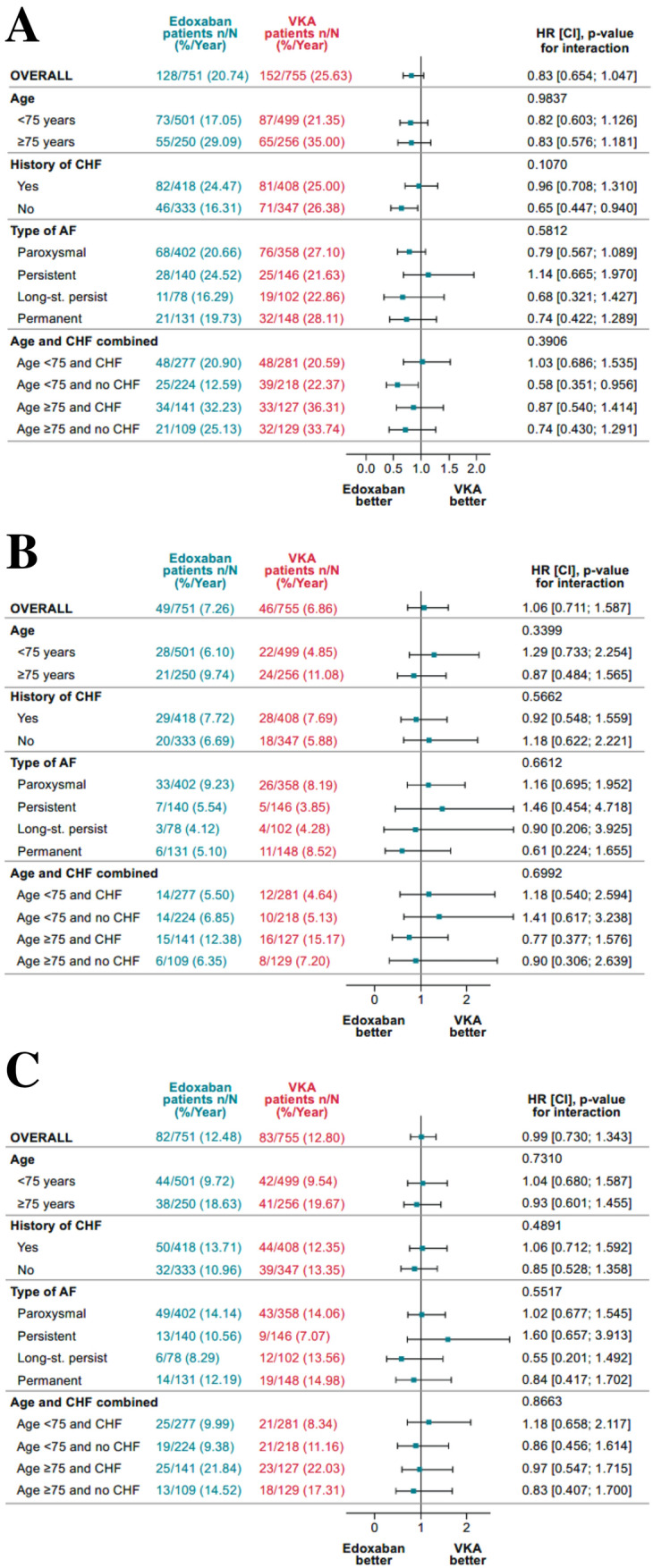
Major or CRNM bleeding (**a**), primary efficacy (**b**), and net clinical benefit outcomes (c) for patients treated with edoxaban- or VKA-based regimen

A recent imaging study has clearly shown that age above 75 years and a history of CHF are the main factors leading to atrial clot formation in AF [10]. Therefore, we analysed these factors in more detail in the PCI setting. Major or CRNM bleeding was not significantly different in patients aged ≥ 75 years compared with those aged < 75 years (Fig. 3a). For patients categorised by age classes (< 75 / ≥ 75 years), history of CHF (yes/no), and the combinations of both parameters, the *p*_interaction_ for major or CRNM bleeding, were 0.98, 0.11, and 0.39, respectively. In patients aged < 75 years and no history of CHF, there was a lower risk of major or CRNM bleeding in those receiving edoxaban versus warfarin.

There was no interaction of age, history of CHF, or combinations of both for the primary composite outcome (Fig. 3b); *p*_interaction_ were 0.34, 0.57, and 0.70, respectively.

**Fig. 4:**
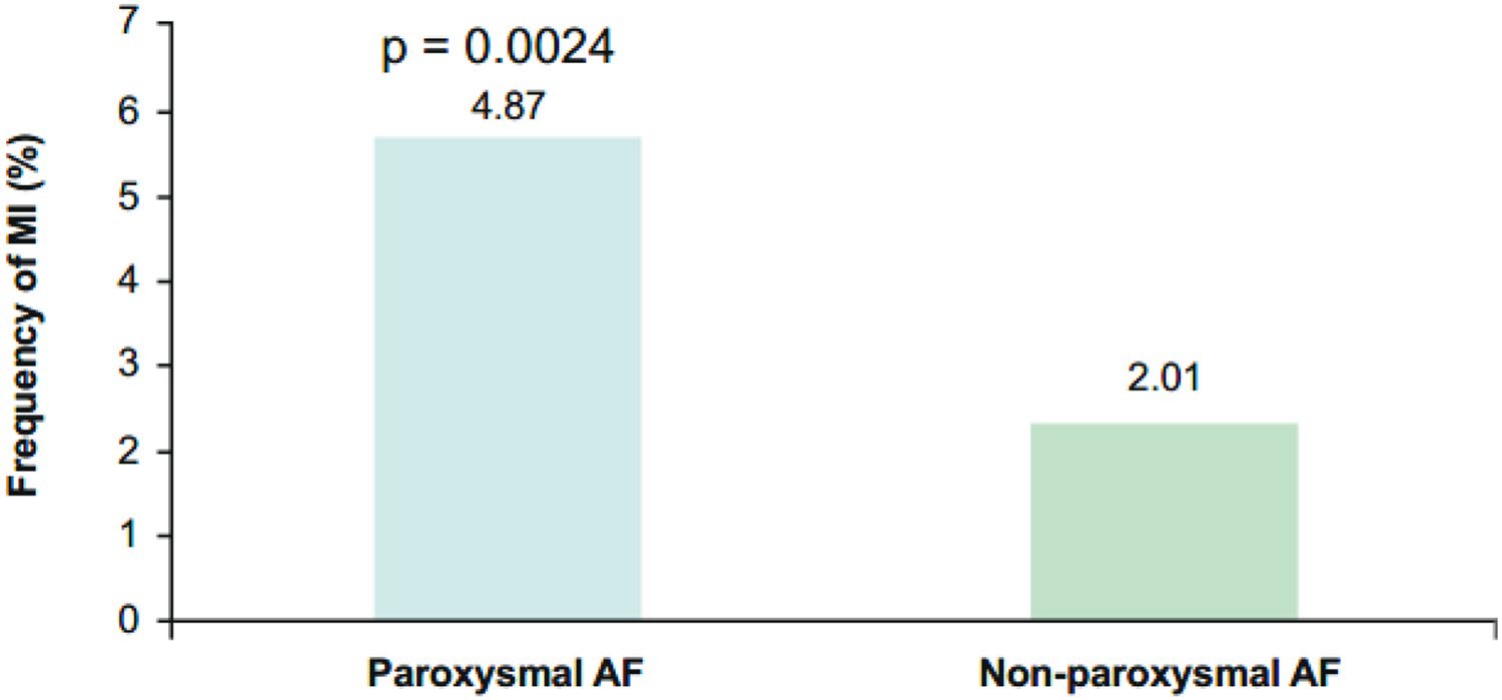
Frequency of myocardial infarction by atrial fibrillation subtype. AF, atrial fibrillation; MI, myocardial infarction

There was no interaction of AF type for major or CRNM bleeding and composite efficacy (Figs. 3a and 3b). In contrast to non-paroxysmal AF, paroxysmal AF at baseline was associated with a higher occurrence of MI (4.87% versus 2.01%, *p* = 0.0024) (Fig. 4, Table 3). Differences in the occurrence of MI cannot be explained by number of stent thromboses (stent thrombosis in patients with paroxysmal AF 30/760, 3.9% versus non-paroxysmal AF 23/745, 3.1%, *p* = non-significant).

**Table 3:** Type of myocardial infarction by the category of atrial fibrillation

Type of MI	Paroxysmal (*N* = 760)	Non-paroxysmal (*N* = 745)	Total (*N* = 1505)^a^
MI type 1: Spontaneous MI	15	8	23
MI type 2: MI secondary to an ischaemic event	6	2	8
MI type 3: MI resulting in death when biomarker values are unavailable	1	0	1
MI type 4b: MI related to stent thrombosis	8	4	12
MI type 4c: MI related to restenosis	7	1	8
Total	37	15	52

MI types 4a and 5 did not occur; both types were adjudicated per definition of the Society for Cardiovascular Angiography and Interventions (SCAI)

^a^1 missing. MI, myocardial infarction

### Net clinical benefit

The event rates of overall net clinical benefit (composite of death from cardiovascular causes, stroke, systemic embolic events, MI, definite stent thrombosis, and major bleeding) were 12.48%/year and 12.80%/year in the edoxaban and warfarin dose groups, respectively (HR [(95% CI]: 0.99 [(0.730; 1.343]) (Fig. 3c).

There was no interaction of age, history of CHF, or combinations of both for the net clinical benefit outcome (Fig. 3c); *p*_interaction_ were 0.73, 0.49, and 0.87, respectively. Furthermore, there was no interaction of AF type for the net clinical benefit (Fig. 3c).

**Fig. 5:**
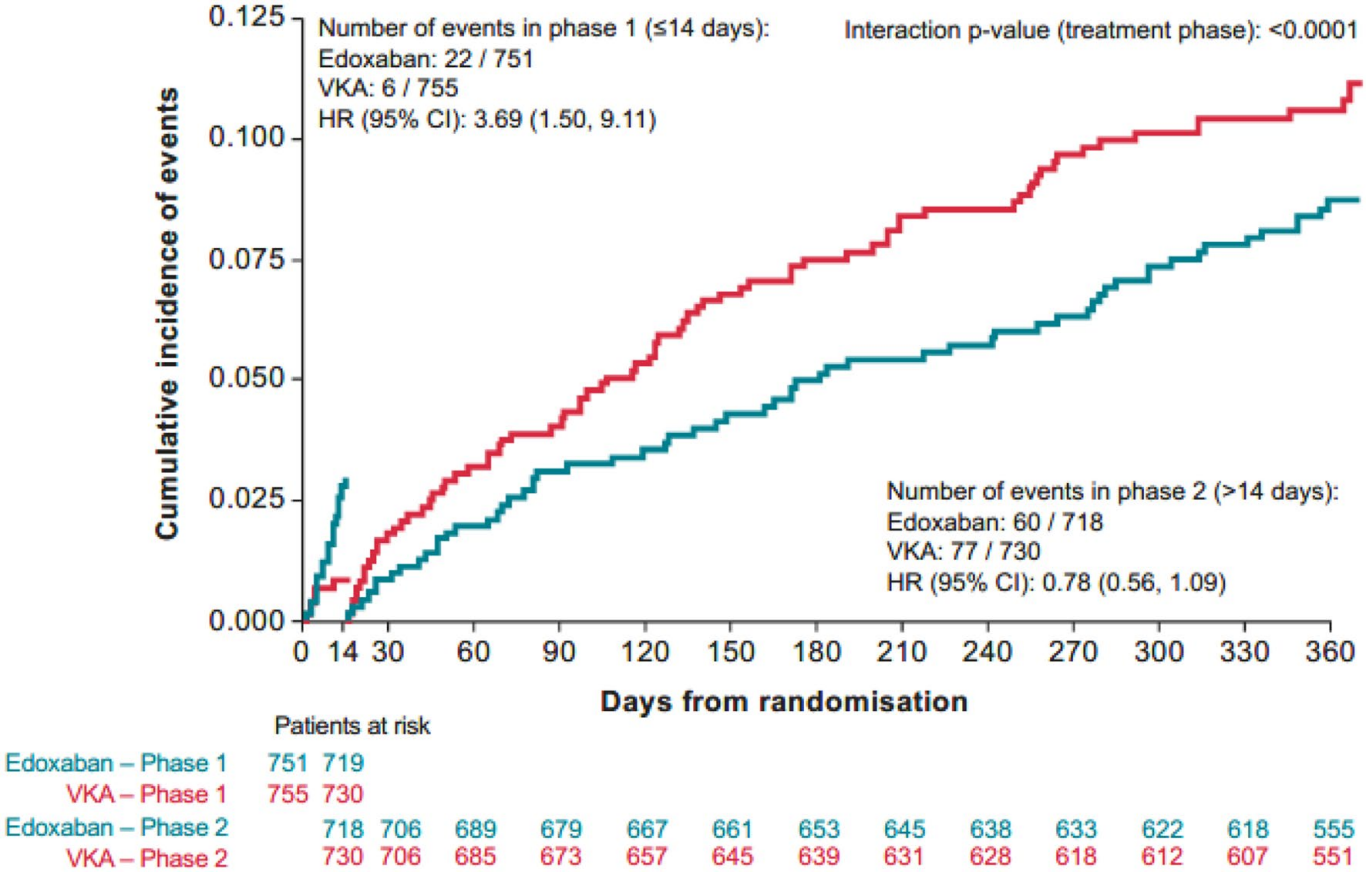
Landmark analysis of net clinical benefit with a landmark set at day 14. Landmark analysis 14 days, 14 days NCB, ITT, overall period + 3 days. *ITT* intention to treat,* HR* hazard ratio, *NCB* net clinical benefit, *VKA* vitamin K antagonist

A post hoc analysis with a landmark at 14 days, and from 15 days to 1 year provided a clear signal of heterogeneity with respect to the net clinical benefit (*p*_interaction_ < 0.0001; Fig. 5).

A lower risk of net clinical composite outcome was noted for the warfarin dose group versus the edoxaban group (HR [95% CI]: 3.69 [1.50–9.11]) in the first 14 days, followed by a lower risk of net clinical composite outcome favouring the edoxaban regimen (HR [95% CI]: 0.78 [0·56–1.09]).

## Discussion

The ENTRUST-AF PCI subgroup analysis showed that the effect of edoxaban versus VKA on study outcomes was consistent independent of CHAD_2_DS_2_-VASc parameters. This is in line with the findings reported in the primary analysis that showed non-inferiority of edoxaban-based regimen compared with the VKA-based regimen for bleeding events, without significant differences in ischaemic events [2].

The coexistence of AF and the need for PCI lead to a higher risk of developing thrombotic and bleeding complications. However, risk stratification in patients requiring dual antithrombotic treatment strategies has not been fully investigated so far. The CHA_2_DS_2_-VASc score was introduced for risk stratification of systemic and cerebral ischaemic events in AF patients. Furthermore, it was shown that this score is useful to predict the overall bleeding risk in AF cohorts [14]. A retrospective study conducted comparative validation of the 6-month GRACE (Global Registry of Acute Coronary Events) risk score and CHA_2_DS_2_-VASc risk score to predict the risk of post-ACS ischaemic stroke [15]. The GRACE risk score was based on nine prognostic variables including age, history of HF, history of acute MI, heart rate, systolic blood pressure, ST-segment depression, serum creatinine levels at admission, elevated myocardial necrosis markers or enzymes, and lack of PCI revascularisation during admission. Both CHA_2_DS_2_-VASc and GRACE risk scores were powerful predictors of stroke incidence during follow-up.

The present analysis suggests that the CHA_2_DS_2_-VASc score is useful to predict bleeding and ischaemic events in AF patients after a successful PCI. Thus, regardless of the treatment regimen of triple or dual therapy, the CHA_2_DS_2_-VASc score is a predictor of adverse clinical outcomes.

In post-PCI patients, stent thrombosis is an event with a substantial risk of death. So far, there is no clinically useful scoring system to predict stent thrombosis in AF patients. The present analysis points for the first time towards the CHA_2_DS_2_-VASc score being a predictor of stent thrombosis in post-PCI AF patients. In patients with CHA_2_DS_2_-VASc scores up to 4, the incidence of any stent thrombosis is in a comparable range as in the AUGUSTUS trial [16]. However, in patients with CHA_2_DS_2_-VASc scores above 4, we observed a marked increase in the risk of stent thrombosis. This new finding may have some key clinical implications. While in the edoxaban group, the vast majority of definite or probable stent thromboses (11/13) occurred within the first 30 days after randomisation, in the VKA group, the majority of definite or probable stent thromboses (8/10) occurred after day 30 following randomisation [17]. Of note, the prediction of total number of stent thromboses with the CHA_2_DS_2_-VASc score was independent of the therapeutic approach with triple versus dual therapy. Overall, our analysis showed that increasing CHA_2_DS_2_-VASc scores were associated with increased events of major or CRNM bleeding, primary outcome, and net clinical benefit in AF patients who underwent PCI.

A landmark analysis for the primary study endpoint has been previously published showing a significant reduction in the rate of the primary bleeding outcome favouring the edoxaban regimen after day 14 [2]. A consistent approach has also been used for the comparison of ACS with chronic coronary syndrome [17]. For the analysis of net clinical benefit with a landmark at day 14, the pattern is similar to the primary study endpoint including a significant heterogeneity with respect to the treatment effect (*p*_interaction_ < 0.0001). However, after the landmark, the net clinical benefit for edoxaban does not reach significance.

The relationship between AF pattern and the risk of adverse outcomes has not been fully explored. A registry demonstrated higher event rates of stroke in persistent versus paroxysmal AF [18]. Furthermore, as noted earlier, the sub-analysis of the ENSURE-AF cardioversion trial pointed towards an increased rate of MI in patients with paroxysmal versus persistent AF [12]. This finding is supported by the present data set, which suggests an increased rate of MI post-PCI in paroxysmal versus non-paroxysmal AF patients.

Pathophysiologically, there are major differences between paroxysmal and persistent AF [19, 20]. Brief episodes of AF induce microcirculatory flow alterations in the ventricles [19]. Thereby, AF induces angina pectoris, low grade of ischaemia characterised by troponin T elevation and a type 2 MI [19, 21]. If a chronic coronary syndrome is already present, such as in all the patients in the ENTRUST-AF PCI trial, an increased and irregular ventricular rate after initiation of AF contributes to impaired blood flow across coronary artery stenosis [21]. At the cellular level, lack of nitric oxide due to the action of reactive oxygen species and oxidative stress appears to be the driver for the flow abnormalities in the ventricular myocardium [19]. Thus, paroxysmal AF has been shown to be associated with ventricular ischaemia in animal models [19, 21]. In contrast to paroxysmal AF, oxidative stress is counterbalanced by downregulation of oxidative stress enzymes and signaling pathways in persistent AF [19]. Thereby, the occurrence of angina pectoris and ventricular ischaemia directly induced by AF is less likely to occur [22]. However, constantly increased ventricular rates during persistent AF patients have been shown to induce a tachycardia-induced cardiomyopathy and heart failure [19, 22].

### Limitations

All retrospective subgroup analyses of clinical trials are subject to many limitations. Such observations should be viewed as hypothesis-generating only. One limitation of this analysis is that the burden of AF or AF recurrences were not systematically assessed during the follow-up period. No constant rhythm monitoring was done. However, 1 year of follow-up may not be long enough to change the pre-existing type of AF. The study was too small to elaborate differences in various treatments regimens (triple versus dual therapy) in accordance to the risk score system. Net clinical benefit was a pre-specified secondary outcome of the study. However, due to the fact that the study did not show a statistically significant difference in the primary endpoint, net clinical benefit is hypothesis-generating and should be interpreted with caution. Moreover, a statistically powered analysis appears difficult due to the overall small events rates. This includes the results regarding stent thromboses which are based on a total of 23 events only for definite plus probable stent thromboses (53 events for any stent thromboses).

## Conclusions

The effect of edoxaban versus VKA in the present cohort was consistent independent of AF-specific stroke risk factors. Of note, increasing CHA_2_DS_2_-VASc score was associated with increased bleeding events and a CHA_2_DS_2_-VASc score ≥ 5 predicted the occurrence of stent thrombosis. Paroxysmal AF at baseline appeared to be a risk factor for MI during follow-up. These findings may have important clinical implications.

## Data Availability

The ENTRUST-AF PCI trial is sponsored by Daiichi Sankyo. Multiple substudies are predefined. Internal investigators, who actively participated in the study, and who provide a methodologically sound study proposal will be granted priority access to the study data for 60 months. After 60 months, access is extended to external investigators not affiliated to the trial. Study proposals can be filed at the Vivli website. For data access requests, see https://vivli.org/.
